# Evaluation of *in vitro* irradiation setup: Designed for the horizontal beamline at the Danish Centre for Particle Therapy

**DOI:** 10.2340/1651-226X.2024.19657

**Published:** 2024-02-13

**Authors:** Anders Tobias Frederiksen, Morten Bjørn Jensen, Per Rugaard Poulsen, Niels Bassler, Brita Singers Sørensen, Mateusz Sitarz

**Affiliations:** a Danish Centre for Particle Therapy (DCPT), Aarhus University Hospital, Aarhus Denmark; b Department of Experimental Clinical Oncology, Aarhus University Hospital, Aarhus, Denmark; c Institute for Biomedicine, Aarhus University, Aarhus, Denmark; d Department for Medical Physics, Aarhus University Hospital, Aarhus, Denmark; e Department of Public Health, Department of Biostatistics, Aarhus University, Aarhus, Denmark

**Keywords:** Radiobiology, proton therapy, water-phantom, Clonogenic assays, irradiation modalities

## Abstract

**Background:**

Radiobiological experimental setups are challenged by precise sample positioning along depth dose profile, scattering conditions, and practical difficulties that must be addressed in individual designs.

The aim of this study was to produce cell survival curves with several irradiation modalities, by using a setup designed at the Danish Centre for Particle Therapy (DCPT) for *in vitro* proton irradiations using a horizontal beam line and thereby evaluating the setups use for *in vitro* irradiations experiments.

**Materials and methods:**

The setup is a water phantom suitable for *in vitro* research with multiple irradiation modalities, in particular the pencil scanning proton beam available from a horizontal experimental beamline. The phantom included a water tank of 39.0 × 17.0 × 20.5 cm. Cell survival-curves were produced using the cell line V79 Chinese hamster lung fibroblast cells (V79s) in biological triplicates of clonogenic assays. Cell survival curves were produced with both a 18 MeV electron beam, 6 MV photon beam, and a Spread-Out Bragg Peak (SOBP) proton beam formed by pristine energies of 85–111 MeV where three positions were examined.

**Results:**

Survival curves with uncertainty areas were made for all modalities. Dosimetric uncertainty amounted to, respectively, 4%, 3% and 3% for proton, electron, and high energy photon irradiations. Cell survival fraction uncertainty was depicted as the standard deviation between replications of the experiment.

**Conclusion:**

Cell survival curves could be produced with acceptable uncertainties using this novel water phantom and cellular laboratory workflow. The setup is useful for future *in vitro* irradiation experiments.

## Introduction

The amount of proton facilities is increasing yearly because of the potential tissue sparing properties using proton irradiation. Setups for preclinical radiobiological experiments at these facilities are most often constructed locally at radiotherapy research centres and rarely shared among more than one proton facility. This makes thorough descriptions and evaluations of each individual setup crucial to ensure scientific reproducibility and transparency of scientific results [1]. Water setups are utilized in numerous dosimetric and preclinical radiobiological experiments where setups are frequently described in methodological articles [1, 2]. The benefits of water in preclinical radiobiological setups include easily controllable surroundings of the cell samples and advantages in dosimetric calculations in treatment planning systems. This is important because radiobiological *in vitro* experiments can be challenging in terms of achieving precise sample positioning along depth dose profile, scattering conditions, and potential practical difficulties that must be addressed in individual designs [1, 3]. A horizontal beamline can be especially tricky because standard dishes for cell culturing cannot be used [4].

The aim of this study was to produce cell survival curves using V79 Chinese hamster lung fibroblast cells (V79s) in a water phantom appropriate for *in vitro* radiotherapy research within the same biological framework using a pencil scanning proton beam available from a horizontal experimental beamline. These experiments were conducted to thoroughly describe and evaluate the radiobiological framework for conducting *in vitro* studies at the Danish Centre for Particle Therapy to ensure reproducibility and transparency for future scientific endeavors conducted with this setup.

## Materials and methods

### Characterization of the phantom

The water phantom of the size 39.0 × 17.0 × 20.5 cm^3^ was constructed with 0.96–0.97 cm acrylic glass (see Supplementary Appendix 1). On top of the phantom, a metal slider could be mounted at 14 firmly fixed positions. Attached below were 3D-printed holders (up to 4 at a time) for inserting of Thermo Scientific^TM^ Nunc^TM^ (T25) cell flasks vertically such that the flasks front walls could be positioned at water depths from 1 to 10 cm. The flasks were additionally fixated with rubber bands or waterproof tape during experiments resulting in a bottom side placement uncertainty of ±1mm evaluated on CT-scans (see Supplementary Appendix 2). The cells were irradiated through the bottom side of the culture flask so that any density-variations in the cell medium would then occur distally from the cell-layer. In this way any such variations during proton irradiations wouldn’t influence the dose in the cell-layer [5]. The very top of the flask cap was not covered with water during the placement in the phantom, thus avoiding contamination through the breathing holes of the filtered cap. The setup can be easily modified by printing a different holder to suit the experimental requirements, for example, different flask or sample rotation.

### Clonogenic assay and analysis of cell survival

The influence of the number of single cells seeded on the plating efficiency (PE) (cellular cooperation) for the V79 cell-line was investigated in accordance with the method described in Brix et al. [6] to ensure that PE-normalization could be used. All cell handling was performed in compliance with the Brix et al. protocol [6]. Every dose of every modality was repeated three times with different passages of biological replicates. Three samples were made per dose and 18 controls total for PE-normalization. Colonies within each biological replicate were fixated and stained at the same timepoint after irradiation using methanol and toluidine blue. SF were then averaged over the three biological replicates. Standard deviation (SD) was calculated based on the three SFs, and standard errors were calculated based on these as suggested by Brix et al. This yielded dose-SF plots at each position in the proton beam as well as for the other modalities. These data were fitted with least square method to the well-established linear quadratic model [7] described in Equation 1:


$$\begin{array}{cc} SF=e^{-\alpha D-\beta D^{2}} & \left(1\right) \end{array}$$ (1)

Equation 1: Linear quadratic model used. *SF* is the survival fraction, *D* is dose, *α* and *β* are fitting parameters.

Further details on cell handling in the experiments can be found in Supplementary Appendix 3.

### Irradiation conditions

#### Water equivalent thickness measurements

For estimating the dose-shift caused by the phantom wall an experiment was designed to measure their water equivalent thickness. The experiment was conducted using a multilayer ionization chamber device (Giraffe, IBA Dosimetry). The Giraffe was placed behind the empty phantom and measured Bragg peak depth for 16 different proton energies spanning from 70 to 220 MeV. Afterward the Bragg peak depths were measured without the phantom in front of the Giraffe. The 80% distal edge position was determined for each irradiation and the shift caused by the two walls of the phantom was estimated for each beam energy. These WET tests yielded a 1.13 cm wall thickness. Confidence intervals of 95% was from 1.12 to 1.14 cm.

#### Irradiation of clonogenic assay samples

**Figure 1:**
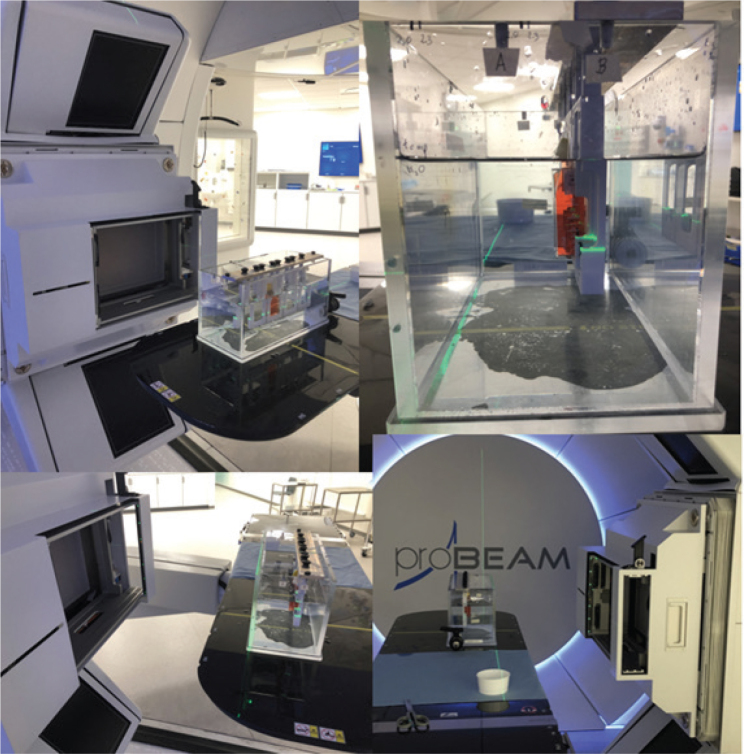
Pictures of the irradiation setup. (More can be found in Supplementary Appendix 7)

The phantom setup is seen in Figure 1, where the phantom is aligned with lasers and a cell-sample filled with medium is placed for irradiation on front the proton gantry. The samples were irradiated with three different modalities: Linear accelerator (LINAC) 6 MV photons, 18 MeV electrons, and mid beam, semi-distal beam, distal edge, and proximal beam Spread-Out Bragg Peak (SOBP) with pencil beam scanning protons with energies from 111 to 85 MeV. During all irradiations the phantom water was kept constant between 20 and 22°C to prevent water cooling from the tank-walls due to lower temperatures in the gantry-room. Each sample was emerged in the tank for around 2 min. Both controls and irradiated samples were exposed to same environmental conditions. Proton, 18 MeV electrons and 6 MeV photon plans were made in Eclipse treatment planning system (TPS) (Eclipse 16.1, Varian Medial Systems, Palo Alto, USA) [8], and proton field uniformity was verified using radiochromic films [9]. Dosimetric uncertainty was thoroughly evaluated for all proton, electron and photon irradiations and were determined to be below, respectively, 4%, 3% and 3%. Descriptions of the irradiation techniques and dosimetric evaluations for each modality are found in Supplementary Appendix 4. Uncertainty of cell survival on the plots in results are the SD of the three repetitions (biological replicates) of the experiments.

## Results

**Figure 2:**
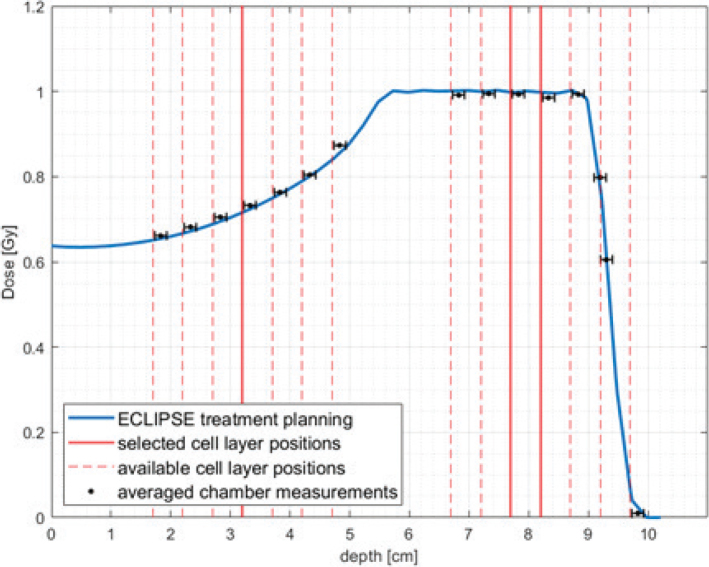
Cell layer positions (red lines) at different depths of SOBP (blue line) calculated in ECLIPSE treatment planning system. The treatment plan used to obtain this data was also used to irradiate cells in the middle of SOBP with the physical dose of 1 Gy (plans for different doses and positions were scaled adequately). Absolute doses were verified with ROOS and Markus ionization chambers (chamber position corresponds to the reference point).

**Figure 3:**
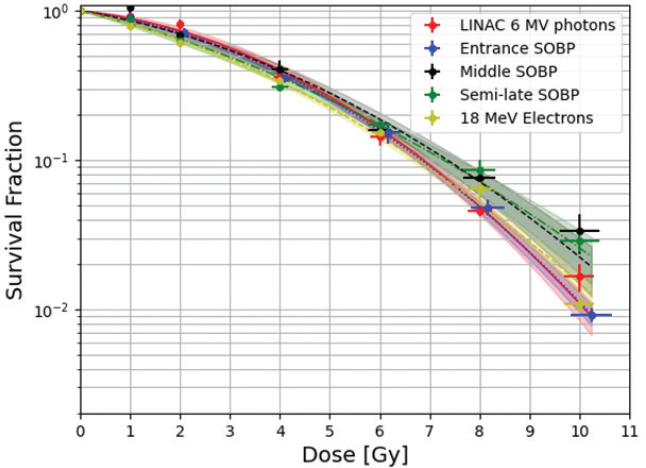
All survival curves in one plot. Three curves for different positions in the proton beam, one LINAC 6 MeV photons survival curve and one 18 MeV Electrons curve. The areas of confidence on the survival curves indicate 1 S.E.

The cellular cooperation experiment yielded that PE-normalization was applicable as V79s did not show signs of cellular cooperation in this setup as seen in Supplementary Appendix 5. Irradiations yielded the fitted data shown in Figure 3. Uncertainty of cell survival on the plot is the SD of the three repetitions (biological replicates) of the experiments, while the uncertainty on the x-axis is dosimetric uncertainty. Samples of the proximal proton irradiation position received a consistently slightly higher dose, shifting the datapoints rightward. More fitted data are seen in Supplementary Appendix 6 where survival fractions of each dose are also found. Irradiations at the distal edge SOBP position were not attempted due to a ±1mm positioning uncertainty (Figure 2) on the flask, which corresponds to around 30% dose error. This was deemed too extensive to report meaningful results. Supplementary Appendix 2 shows CT-scans of the phantom picturing this placement uncertainty.

## Discussion

The primary finding is the demonstration of a convenient setup and workflow for *in vitro* irradiations using a horizontal beamline yielding acceptable dosimetric uncertainties using this novel water phantom. Cellular cooperation is not a concern with V79 Chinese hamster lung fibroblasts in this setup, therefore PE-based normalization was fair to use. When comparing cell survival curves for a variety of radiation modalities it comes as no surprise that these differ slightly since the relative biological effectiveness (RBE) of these modalities are different [10]. This influences both overall effectiveness of the irradiation modality as well as dose dependent cell-kill. The setup is useful for irradiation in multiple modalities and has a size that makes photon dose-plans such as 15 × 15cm used on patients transferable. Multiple biological replicates can be irradiated simultaneously in the same layer, allowing for quick and reproducible cell survival curves, and finally a great advantage with this setup is that the cells are positioned distally from known homogenous and callable mediums. The protons meet first phantom wall, then water and the finally plastic bottle in their trajectories before hitting the cells. This contrasts to existing setups using multiple plastic walls with cells and cell-medium in between them resulting in a heterogenous sequence of targets, which might prove difficult to calibrate [4]. The phantom is useful for high energy photon, electron and proton irradiation experiments as illustrated.

The water-phantom is advantageous in terms of controlling the temperature during irradiations. While *in vitro* experiments are often kept at 37°C throughout the experiment, this was prioritized differently during the clonogenic assay. Maintaining water-temperatures deviating room temperature by multiple degrees is in risk of being less consistent due to air-cooling of the walls, affecting the water density and thus the dose at cell level. Instead, water temperature was kept consistently between 20 and 22°C to ensure the same water density on different biological replicates. Since the irradiated samples in this setup is normalized to controls within each biological replicate, temperature shifts from the environment would be reflected in all samples including controls, and thus the primary concern is uniformity of water temperature in the irradiation field.

The use of clonogenic assays have been widely employed in the field of radiobiology. One should be attentive of the limitations while interpreting cell survival curves. For example, technical replicates are important to estimate the uncertainties of the workflow itself. When counting colonies, a cutoff value of 50 cells per colony is typically used. Using a cutoff value of 50 cells risks disregarding important biological information on the growth of the colonies such as colony shape, spatial growth rate over time or colony-size [11]. Future studies could benefit from exploring alternative methods for radiation modality comparison, which may be better suited for capturing the full range of damage from radiation on cells and tissues. As an example, Koch et al. [11] have demonstrated characterization of the temporal development of colony size and fractions of differential growth behavior.

Irradiating through the bottom side of the flask placed the cell-layer proximately from any possible density-variations in the cell medium. This yielded a different consideration: variations of the cell flask plastic might influence the shift slightly and prevent the cells from being in charged-particle equilibrium. This could occur if stray electrons from the plastic were knocked loose by the primary beam. The dose influence of stray electrons is believed to be smaller than the risk of dose-shift by density variations in the cell medium. The gain from dose certainty by irradiating through the bottom side is believed to be higher than the effects of a possible charged-particle in-equilibrium.

Based on this study, the phantom is very useful for conducting *in vitro* irradiation studies, using both high energy photons and protons. Other modalities might likewise be compatible with the phantom, but this might require additional setup descriptions and adjustments. Dosimetric uncertainties were thoroughly investigated and found to be 3%, 3% and 4% for high energy photons, electrons and protons creating base for making cell-survival curves with acceptable brims of uncertainty.

## Supplementary Material

Evaluation of *in vitro* irradiation setup: Designed for the horizontal beamline at the Danish Centre for Particle Therapy

## Data Availability

Data available for sharing to anyone interested by contacting corresponding author.
