# Supplementary material for: Evaluation of in vitro irradiation setup: Designed for the horizontal beamline at the Danish Centre for Particle Therapy
Source: Acta Oncol. 2024 Feb 13;63:19657. doi: 10.2340/1651-226X.2024.19657 (PMC11332460; doi:10.2340/1651-226X.2024.19657)
Supplement: Evaluation of in vitro irradiation setup: Designed for the horizontal beamline at the Danish Centre for Particle Therapy [file AO-63-19657-s1.pdf]

Supplementary material has been published as submitted. It has not been copyedited or typeset by Acta Oncologica.

Supplementary reading

Appendix 1 Phantom CT-scans and Pictures

Measurements from the CT-scans were verified using rulers and calipers.

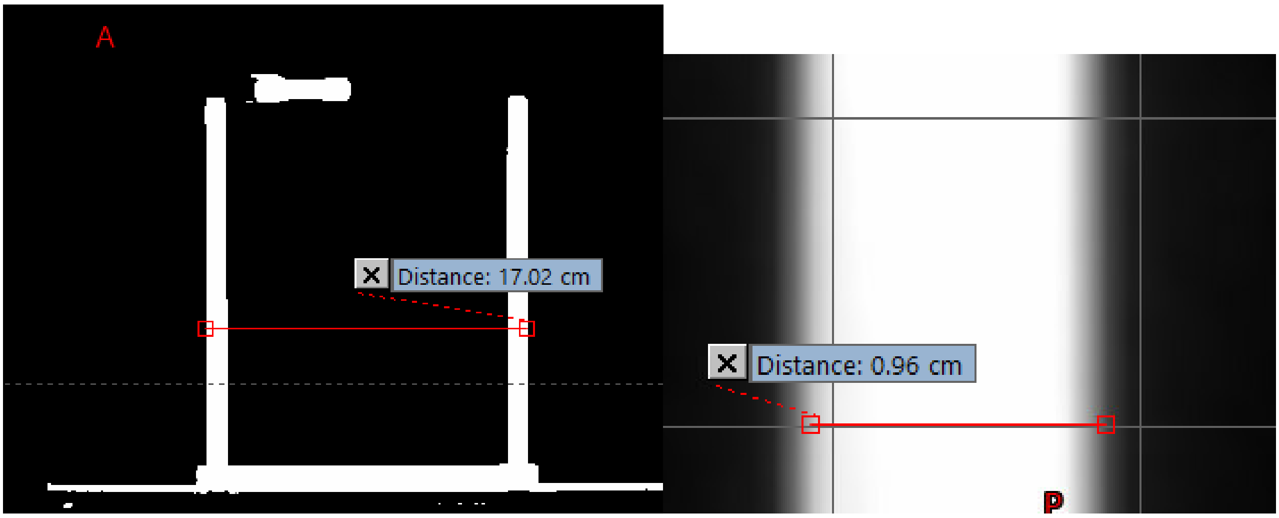

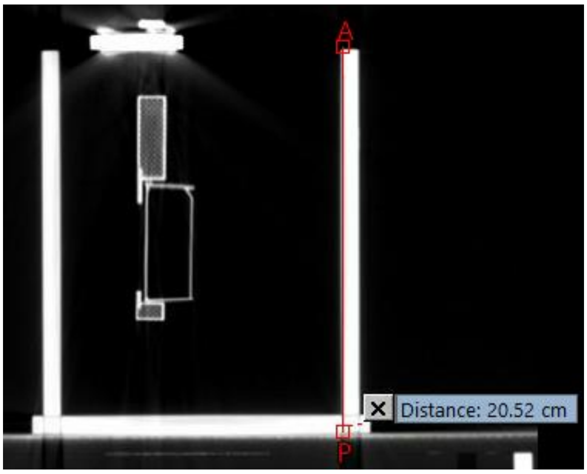

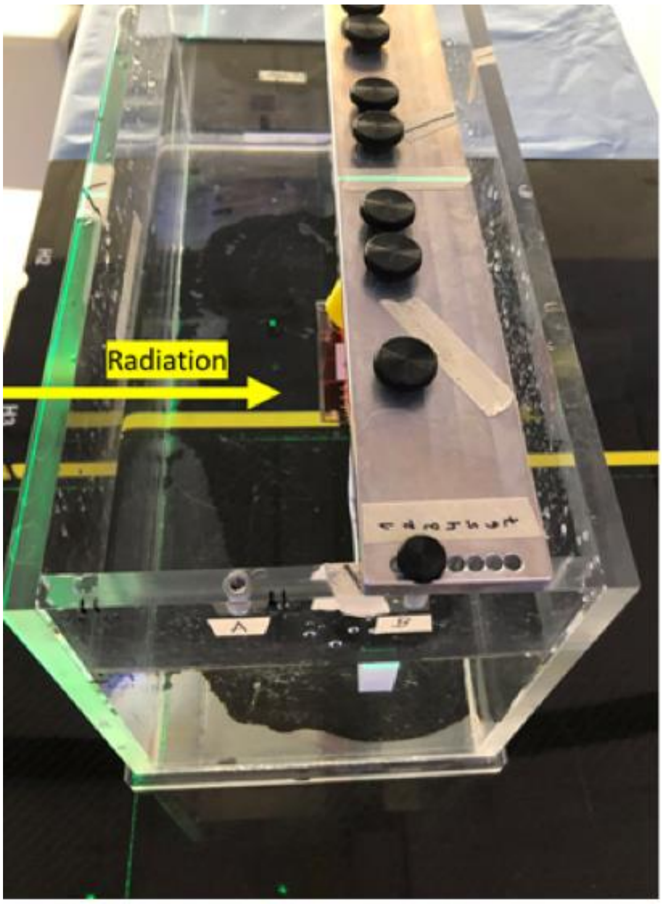

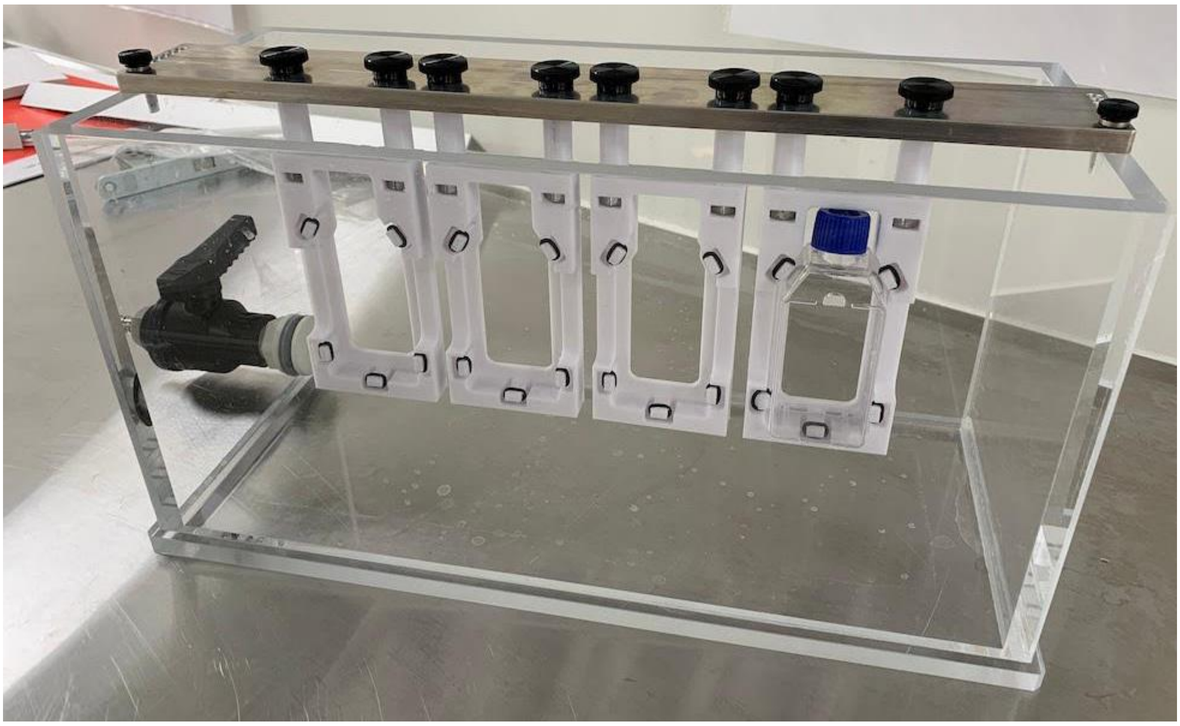

Appendix 2 Placement uncertainty evaluated on CT-scan.

Pictures are 5 continuous CT-voxels taken vertically indicated with the red line with 0,6mm distance in between each picture in the direction shown with the blue arrow indicated on the colored picture below. Starting from the top CT scan picture its seen that the upper right corner is closer to the beam-entrance wall, thus the first cells would be meet on the third picture once through the flask plastic. It takes two more voxels before the CT-scan displays complete clearance of the plastic. This displays how the cells meet the beam unevenly yielding an approximate 1mm placement uncertainty.

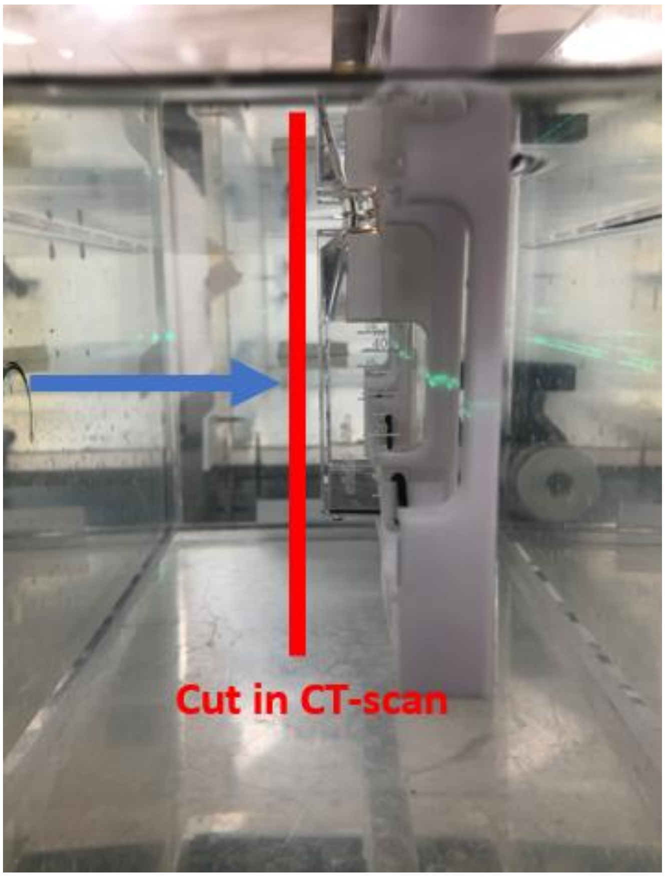

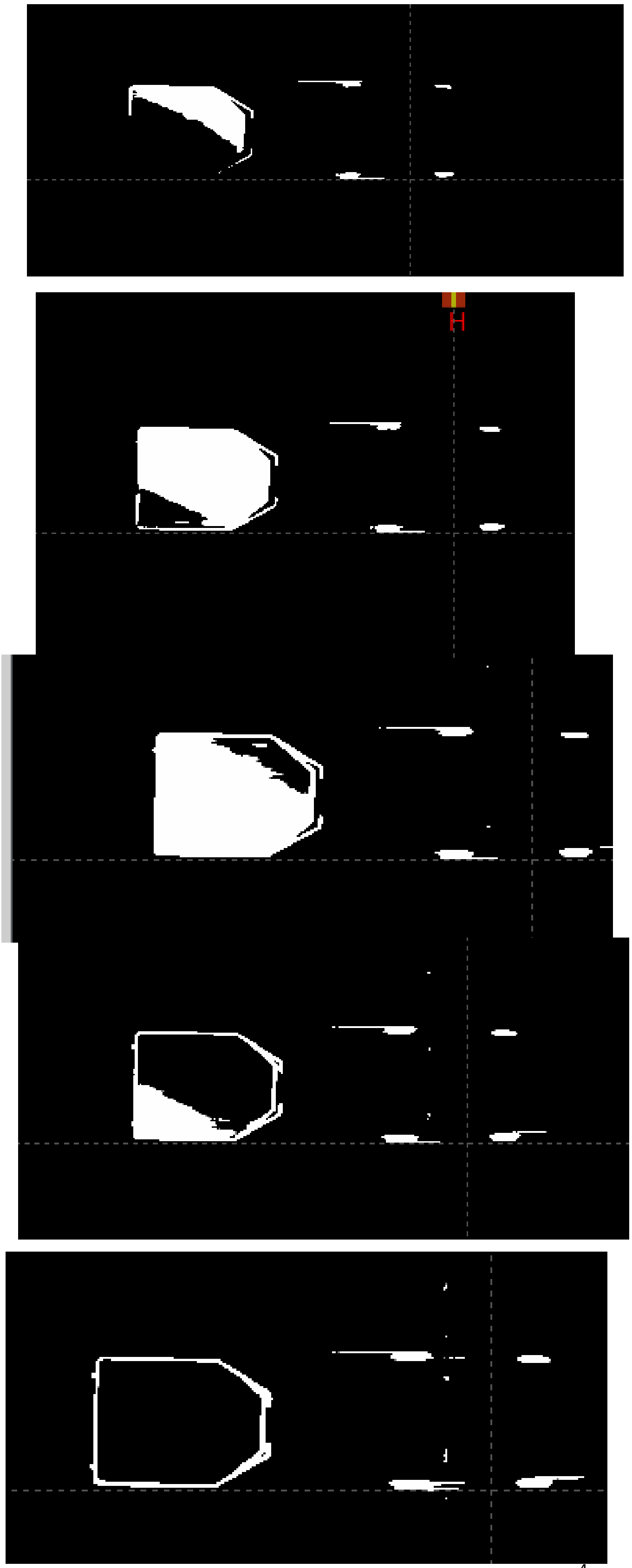
H

Appendix 3 – Details on cell conditions

For all experiments V79s were cultured in Thermo Scientific™ Nunc™ T75 culture flasks and transferred to Thermo Scientific™ Nunc™ T25 flasks for irradiation aiming for 120 colonies after the final incubation period in the experimental samples. These flasks were equipped with filter-caps that ensured 5% CO2 within the flasks while preventing contamination when removed from the incubator. Samples were then incubated for 6 to 8 hours in horizontal position prior to irradiation. The same culture medium (MEM, 10% FBS, 1% p/s, 1% NEAA, 1% SP) was used throughout the experiments. Colonies were counted with the standard threshold of 50 cells per colony. After counting all irradiated flasks, survival fractions (SF) were calculated on each individual flask using Equation 1:

$$SF = \frac{C}{\mathrm{S}}$$

Equation 1: Survival fractions. C was obtained colonies in irradiated flasks normalized to seed out volume. S was average count of cells in the 6 controls (reference flask on each biological replicate) normalized to seed out volume in controls.

Appendix 4– Irradiation techniques

LINAC 6 MV photons and 18 MeV electrons

LINAC 6 MV photons and 18 MeV electrons irradiations were caried out using a 100 cm source-to-axis distance TrueBeam linear accelerator (Varian Medial Systems, Palo Alto, USA). The setup was CT scanned (Brilliance CT BigBore, Philips Medical Systems, Cleveland, USA) with a 0.5 mm slice thickness and imported for treatment planning. A reference point was chosen to coincide with the centre of mass of the cell-layer, and the number of monitor units (MU) needed to deliver a certain dose was calculated by normalizing the treatment plan to deliver 100% prescribed dose in the reference point. For 6 MV photon irradiations, the cell layer was positioned 2.0 cm from the outer wall as measured along

the beam direction, and the treatment was planned with a gantry rotation of 90 degrees, a 10 cm x 10 cm jaw-defined field, 100 cm source-surface-distance (SSD), a flattened 6 MV beam quality and a machine-set dose rate of 600 MU/min corresponding to a dose rate at the cells of approximately 6.4 Gy/min. For electron irradiations, the cell layer was positioned 2.3 cm from the outer wall as measured along the beam direction and the treatment was planned with a gantry rotation of 90 degrees, a 10 cm x 10 cm aperture-defined field, an SSD of 97.7 cm, an 18 MeV beam quality and a machine-set dose rate of 600 MU/min corresponding to a dose rate at the cells of approximately 6.3 Gy/min. Beam quality and distance from outer wall were chosen to optimize robustness of the setup and dose uniformity. Prior to conducting the experiments, the machine output factor was determined following institutional guidelines using a farmer chamber (FC65-G, IBA Dosimetry GmbH, Schwarzenbruck, Germany) and the number of MU needed to deliver a certain dose was corrected accordingly. The setup was aligned with the cell layer in sagittal orientation and centered in the field using the in-room treatment lasers by measuring the distances, as given from the TPS, from the sagittal, transversal, and frontal lasers (supine, head-first coordinate system) to the outer walls of the phantom. Samples received the prescribed dose (as seen in appendix 6) with a total dose uncertainty of +/- 3% including calibration uncertainties of measurement chambers. Pictures of the setup, and the simulated plan can be found in Appendix 7.

111-85 MeV Protons.

Proton irradiations were performed at the experimental fixed horizontal proton beamline with pencil beam scanning at The Danish Centre for Particle Therapy (DCPT) (ProBeam, Varian, a Siemens Healthineers company, Palo Alto, CA, USA). The beam energy range was 111-85 MeV. Flasks were placed in the water, with the cell layer at the water-equivalent depth of 3.2 cm (proximal), 7.7 cm (middle SOBP), or 8.2 cm (semi-distal SOBP). Flasks were irradiated one at a time, with the flask wall positioned in the middle of 9x9 cm^2 uniform field (+-1%). The alignment of the flask relative to the isocenter was verified by visual inspection of irradiated EBT-3 gafchromic film taped to the flask wall.

Prescribed doses were verified with the use of Roos- and Advanced Markus-ionization chambers, taped inside an empty cell flask (rail with flask containing Advanced Markus ionization chamber was aligned without fixating the slider with screws to match the reference point of Roos). The overall uncertainty of prescribed doses was found to be below 4% (not shown on the plot). Flasks were placed so that the beam entered the flask bottom, hit the cells, and continued through the cell medium. This was done under the assumption that the flask plastic varied less between flasks and between biological replicates, than the density of cell medium. Therefore, this methodology was chosen to ensure that cells received the same dose despite daily variations in the cell medium. The setup is seen in Appendix 7.

Appendix 5 – Cellular cooperation results

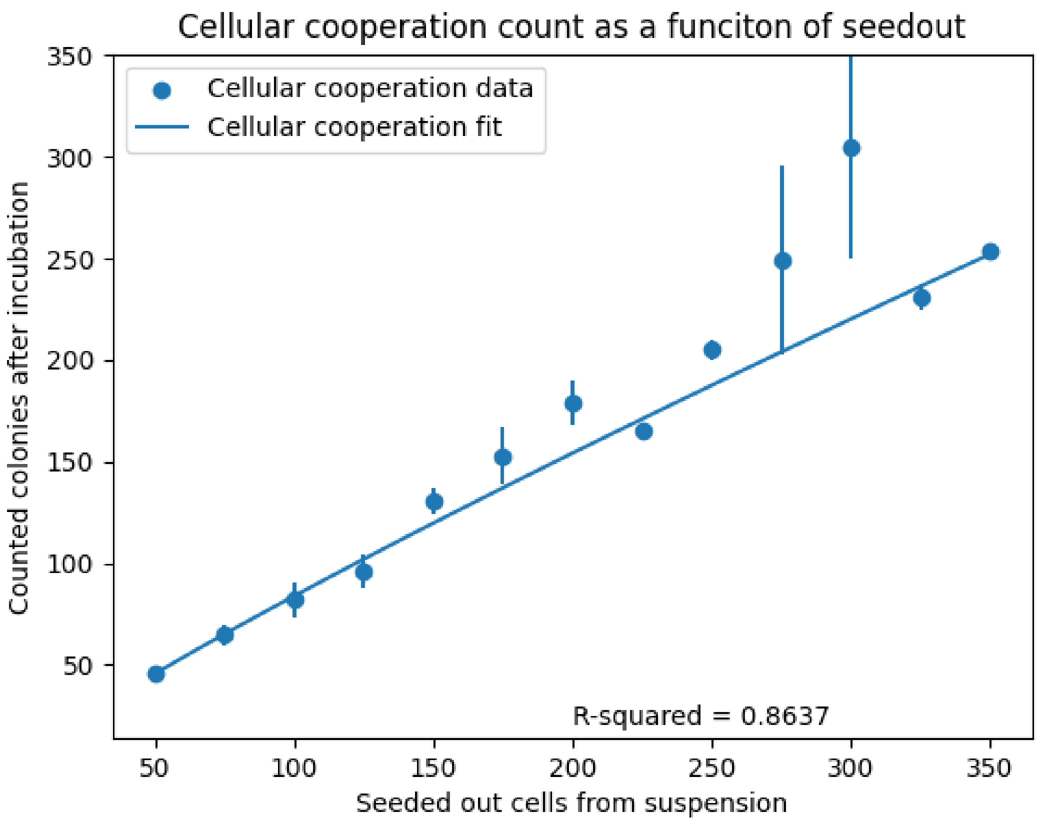

The curve is an exponential fit based on equation 1. Error bars are displaying SD of three technical replicates.

Shows the colony count as a function of seed out volume and using the fit in the following equation:

$$C = a \cdot S^b$$

Cellular cooperation model (Brix et al.); C is the counted colonies after incubation, S is the numbers of cells seeded out, a and b are fitting parameters.

A “b” value of 0.88 and an “a” value of 1.45 was calculated with a reasonable fit yielding a R-squared of 0.86. The b-value referred to is used for assurance that PE-based normalization can be used if it is within the set boundaries of $0.8 < b < 1.2$ given by the Brix et al. (2021) protocol. Thus, the impact of cellular cooperation in this setup was of minor importance and PE-based normalization could be used and the survival curves were made this way as seen in figure 3.

Appendix 6 Survival Fraction Data of Clonogenic Assays

**Position**	**Dose (+- 1 S.E.) at 10% Survival [Gy]**
6 MV photons (LINAC)	6.85 ± 0.63
Proximal position protons	6.83 ± 0.27
Middle SOBP	7.36 ± 0.89
Semi-distal SOBP	7.28 ± 0.91
18 MeV Electrons	6.75 ± 0.38
**Protons - SOBP positions**	**Survival curve fit**
Proximal	$y = e^{-0.1014*D-0.03456*D^2}$
Middle	$y = e^{-0.1329*D-0.02463*D^2}$
Semi-Distal	$y = e^{-0.1924*D-0.01693*D^2}$
**Photons**	**Survival curve fit**
6 MV photons LINAC	$y = e^{-0.07873*D-0.03727*D^2}$
**Electrons**	**Survival curve fit**
Electrons	$y = e^{-0.1768*D-0.02434*D^2}$

Dose /Gy	Photons LINAC SF	Middle SOBP SF	Semi-Distal SOBP SF	Electrons SF
0	1	1	1	1
1	0.92	1.1	0.89	0.79
2	0.81	0.69	0.63	0.61
4	0.36	0.41	0.31	0.34
6	0.14	0.16	0.17	0.15
8	0.046	0.076	0.086	0.065
10	0.017	0.033	0.029	0.011

Dose /Gy	Proximal SOBP SF
0	1
1	0.91
2.08	0.71
4.08	0.35
6.16	0.15
8.16	0.048
10.24	0.0092

Appendix 7 – Pictures of Experimental Setups

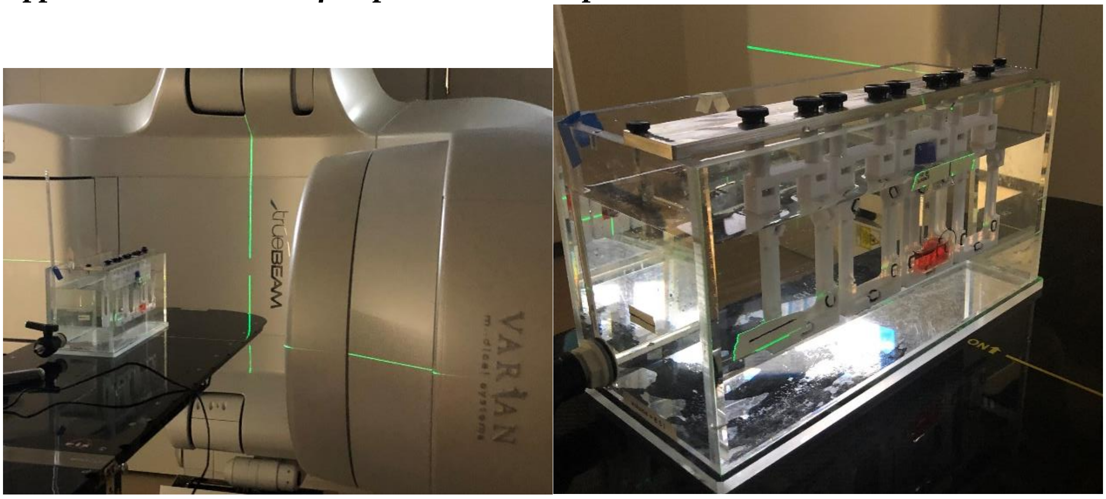

Setup for 6 MeV LINAC irradiations.

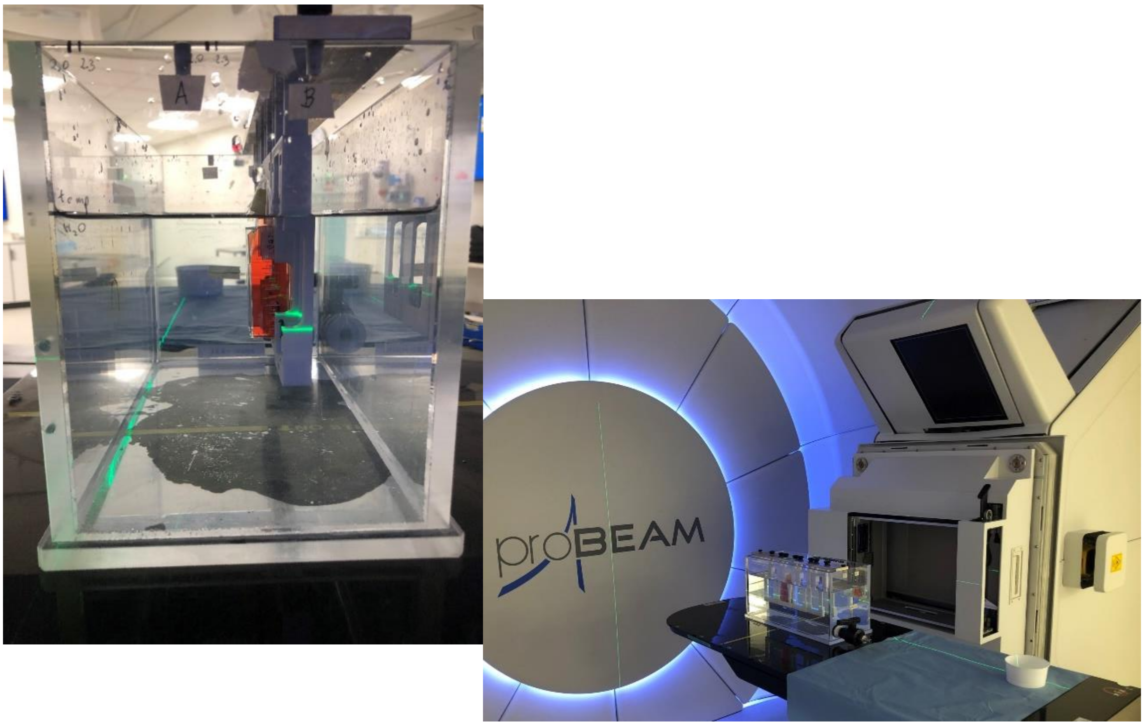

Setup for 85-111 MeV proton irradiations

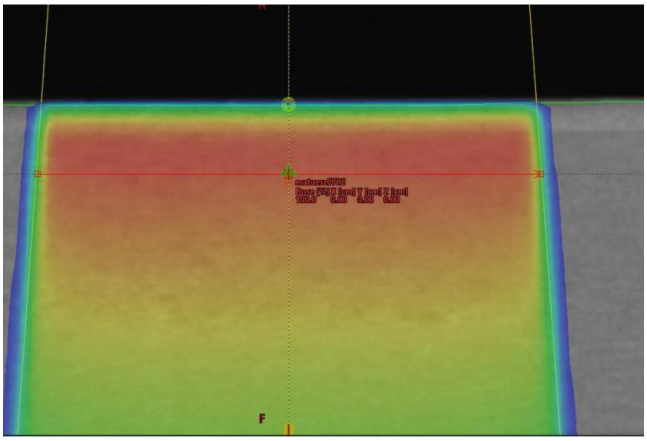

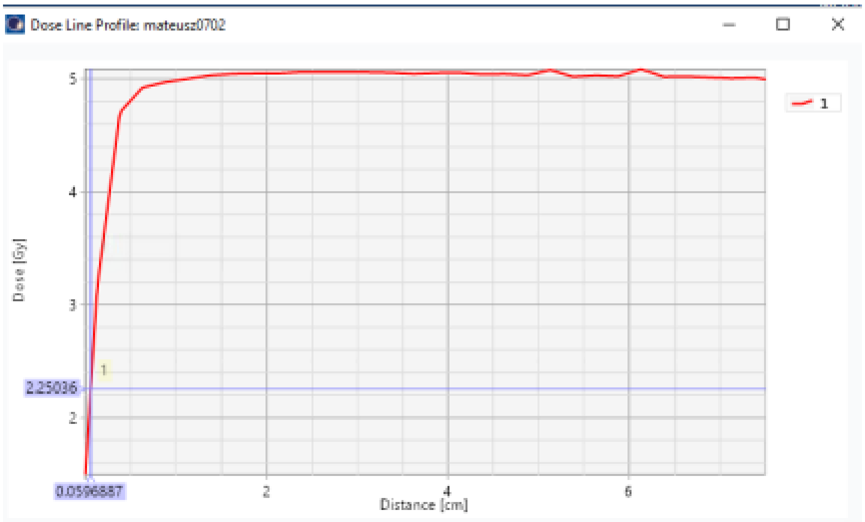

LINAC irradiations dose uniformity in depth

Dose uniformity through the phantom, up being the front of the phantom. Since cells are placed on 2cm depth the uniformity is assured at this point as seen in depth dose profile just below.
